# Effects of Olive Leaf Extract on Anxiety Symptoms and Metainflammation in Women with Excess Weight: A Randomized Double-Blind Placebo-Controlled Pilot Trial

**DOI:** 10.3390/life16071081

**Published:** 2026-06-28

**Authors:** Mario Hernández-Garibay, David Fernández-Quezada, Joaquín García-Estrada, Ulises de la Cruz-Mosso, Rosa Yaveth Ruvalcaba-Delgadillo, Rocio Elizabeth González-Castañeda, Sonia Luquin

**Affiliations:** 1 Instituto de Neurociencias Traslacionales, Departamento de Neurociencias, Centro Universitario de Ciencias de la Salud (CUCS), Universidad de Guadalajara (UdeG), Sierra Mojada 950, Guadalajara 44340, Mexico; 2 Laboratorio de Microscopia de Alta Resolución, Departamento de Neurociencias, Centro Universitario de Ciencias de la Salud (CUCS), Universidad de Guadalajara (UdeG), Sierra Mojada 950, Guadalajara 44340, Mexico

**Keywords:** olive leaf extract, metainflammation, overweight, obesity, inflammation, anxiety, anxious symptomatology

## Abstract

Anxiety symptomatology and excess weight are associated with chronic low-grade inflammation. Olive leaf extract (OLE) contains polyphenols with antioxidant and anti-inflammatory properties that have shown anxiolytic-like effects in experimental models; however, evidence in humans remains limited. This randomized double-blind placebo-controlled pilot trial evaluated the effects of OLE supplementation on anxiety symptomatology, inflammatory markers, and metabolic parameters in women with excess weight and mild to moderate anxiety symptoms. Participants received OLE (750 mg/day) or placebo for 12 weeks. Anxiety symptomatology was assessed using HAM-A, BAI, and STAI, while inflammatory and metabolic parameters were evaluated at baseline and post-intervention. Since the expected sample size was not achieved, our results are preliminary. No effect was observed from OLE supplementation. However, some differences within groups can direct future researchers interested in evaluating these parameters. OLE supplementation correlated with a particular reduction phenotype in anxious symptomatology (HAM-A, BAI and STAI-Trait) with inflammatory (hs-CRP) and metabolic parameters (c-HDL) that may open the possibility of an anti-inflammatory effect. In contrast, the observations in the Pb group seemed more associated with a pro-inflammatory state and a placebo effect related to somatic symptoms of anxiety. These preliminary observations need to be confirmed with larger randomized clinical trials that may help clarify these results and the possible underlying mechanisms.

## 1. Introduction

The most prevalent mental health disorders are those related to anxiety, with a worldwide prevalence of 4.4% [1]. Anxiety is a specific emotional state caused by a potentially dangerous situation [2]. Generalized anxiety disorder (GAD) consists of an excessive, multifocal and difficult-to-control worry. People with GAD show intolerance of uncertainty (negative reaction to uncertain situations). The diagnosis depends on the focus of worry. It is associated with illness (health anxiety disorder), irrational beliefs (compulsions), interaction or performance with other people (social anxiety), abrupt unexpected fear and physical symptomatology (panic disorder) or history of life-threatening trauma (post-traumatic stress disorder) [3]. Anxiety can also be categorized into state anxiety and trait anxiety. State anxiety is characterized by an acute cognitive-worry (self-doubt and potential failure) and an emotional arousal (stress-sympathetic) response to the potential threat. Meanwhile, trait anxiety refers to a predisposition to present state anxiety in a specific situation [2,4].

Anxious behavior is related to brain regions like the amygdala, hippocampus and hypothalamus [5]. During emotional arousal there is a stress response: the amygdala stimulates the brain stem and promotes catecholamine liberation, increasing heart rate and respiration and producing an immune proinflammatory response to deal with antigens and possible foreign menaces. It also releases corticotropin-releasing hormone (CRH) to consequently synthetize cortisol through the Hypothalamic–Pituitary–Adrenal (HPA) axis. Cortisol promotes glucose mobilization for fuel and an anti-inflammatory reaction to cope with the initial response. With chronic stress, excessive cortisol and inflammation alter the anti-inflammatory cortisol function by disrupting its receptor activity [6].

Thus, inflammation is closely related to anxiety. Stress production of proinflammatory cytokines like TNF-α and IL-6 have been shown to alter blood–brain barrier (BBB) permeability, favoring translocation of immune cells and other proinflammatory molecules that activate astrocytes and microglia, promoting central inflammation [7]. In the brain, proinflammatory cytokines can stimulate the HPA axis to synthetize CRH and consequently cortisol [8,9]. In the amygdala, CRH augments NMDA activity, while Ca^+2^ influx and brain-derived neurotrophic factor (BDNF) strengthen neuronal communication. In contrast, in the hippocampus, CRH reduces BDNF levels, shortening neuronal communication. These neural changes contribute to HPA axis overstimulation since the amygdala has inhibitory and the hippocampus excitatory projections to inhibitory neurons in the paraventricular hypothalamic nucleus (PVN) [10]. By these mechanisms, anxiety can become chronic.

Another process associated with inflammation is excess weight (overweight and obesity). In normal conditions, visceral adipose tissue (VAT) contains immune cells that regulate its function [11]. With excess weight, adipocytes increase in number and size [12]. This produces a hypoxic environment that promotes oxidative stress, inflammation and cellular death, inducing the recruitment of more immune cells, mainly macrophages, and exacerbating the inflammatory processes by pathways like the activation of toll-like receptors (TLRs), production of pro-inflammatory (TNF-α, IL-6) and reduction in anti-inflammatory cytokines (IL-10) [11,13]. This metabolic inflammation process is referred as metainflammation [13].

It has been observed in young adults with excess weight have higher plasma levels of macrophage attractant chemokines CCL2, CCL5 and CXCL16 [14]; IL-6, high-sensitivity C-reactive protein (hs-CRP) [15]; and hormones like resistin and leptin [14]. Some studies have reported higher levels of C-reactive protein (CRP) and leptin in women than in men [16].

Inflammation disrupts insulin and leptin signaling [17]. Insulin receptor (IR) knockout (KO) in the hypothalamus is associated with anxious behavior [18]. Cortisol also promotes leptin resistance [19], whose correct function is associated with anxiolytic effects through the ventral tegmental area (VTA) [20].

Women have two times more probability of being diagnosed with an anxiety disorder [21]. Obesity increases the probability of an anxiety disorder by 30% and anxiety symptoms by 40%; the diagnosis of an anxiety disorder is nearly double for obese women compared to obese men [22]. The excess weight prevalence in Mexico is 76.8% for women (40.2% obesity; 36.6% overweight) and 73% for men (30.5% obesity; 42.5% overweight). In Jalisco, similar numbers are observed with 75.9% women (39.2% obesity; 36.7% overweight) and 73.1% men (29.5% obesity; 43.6% overweight) having excess weight [23]. As for anxiety, amongst adults across the country: 19.3% present severe (23.2% women; 15% men) and 31.3% mild (32.8% women; 29.7% men) anxiety symptomatology [24]; in Jalisco, anxiety is the most prevalent mental disorder and presents a higher prevalence in women [25].

In recent years, researchers have found beneficial effects in mental health disorders from polyphenol-rich compounds [26]. Olive leaves have shown greater polyphenol content than olive oil or fruit [27]. The principal polyphenols found in the leaves are oleuropein, tyrosol, hydroxytyrosol and verbascoside, among others; their main beneficial effects are associated with a strong antioxidant and anti-inflammatory capacity [27,28].

An anxiolytic effect in murine models has been shown. OLE or oleuropein alone can improve serotonin levels and antioxidant activity and reduce TNF-α and apoptosis markers in the hippocampus, inhibiting anxious behavior [29,30,31]. These beneficial effects in anxious behavior and antioxidant activity in mouse brain have been supported with oleuropein alone in a major depressive disorder model [32].

In humans, there is no study of the possible anxiolytic effects from the leaves. Some of the observed benefits are reduced levels of IL-6, TNF-α, and IL-8 [33] and improved blood pressure [34] and lipid profile [35,36] as well as leptin, adiponectin and free fatty acid levels [37], HbA1C and fasting insulin [38].

Based on this information we decided to evaluate anxious symptomatology, inflammatory markers (TNF-α, IL-6, and hs-CRP) and hormones associated with anxiety (cortisol), and metabolic parameters (total cholesterol, c-HDL, triglycerides, c-LDL, insulin and leptin) related to excess weight in women from Guadalajara Metropolitan Area (GMA), Jalisco, Mexico, supplemented with a commercial olive leaf extract and compared it against placebo to analyze a possible effect in reduction in anxious symptomatology from OLE in human subjects.

## 2. Materials and Methods

### 2.1. Ethical Approval and Study Registration

This study was conducted in accordance with the Declaration of Helsinki and approved by the Research and Ethics Committees of the Centro Universitario de Ciencias de la Salud (CUCS), Universidad de Guadalajara (Approval Number: CUCS/CINV/0061/24). The trial was prospectively registered at ClinicalTrials.gov (NCT06485349). Prior to enrollment, all participants received a detailed explanation of the study objectives and procedures and provided written informed consent.

### 2.2. Study Design

A randomized, double blind, placebo-controlled pilot trial was conducted to evaluate the effects of OLE supplementation on anxiety symptomatology, inflammatory markers, and metabolic parameters in women with excess weight. Participants were recruited between October 2024 and November 2025 through advertisements distributed across the Centro Universitario de Ciencias de la Salud (CUCS), Universidad de Guadalajara, and through digital social media platforms, including WhatsApp, Facebook, and Instagram. Interested participants initially completed a digital self-report screening questionnaire assessing demographic information, anthropometric characteristics, medication use, nutritional supplementation, alcohol consumption, psychiatric history, and chronic disease diagnoses. Subsequently, eligible individuals attended an in-person interview where the information was verified, and baseline assessments were performed.

**Figure 1:**
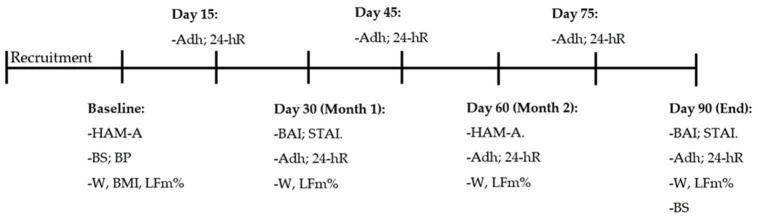
Experimental design timeline. HAM-A, Hamilton Anxiety Scale; BS, blood sample; BP, blood pressure; W, weight; BMI, Body Mass Index; LFm%, lean and fat mass percentage; Adh, adherence; 24-hR, 24 h nutritional recall; BAI, Beck Anxiety Inventory; STAI, State-Trait Anxiety Scale.

At baseline, anthropometric measurements, blood pressure, body composition, and anxiety scales were evaluated. Venous blood samples were also collected before intervention initiation. Participants were then assigned to receive either OLE or placebo supplementation for 12 weeks. Follow-up evaluations were conducted every 30 days, while adherence and dietary recalls were monitored biweekly throughout the intervention period. Final biochemical and psychological evaluations were performed after completion of the intervention. To minimize circadian variability and measurement bias, post-intervention blood samples were collected under standardized fasting conditions within 1–5 days after the last capsule intake according to participant availability (Figure 1).

### 2.3. Participants

Participants meeting the following inclusion criteria were enrolled: women aged 18–40 years from the Guadalajara Metropolitan Area (GMA), with a Body Mass Index (BMI) between 25 and 34.9 kg/m^2^, mild to moderate anxiety symptomatology defined as a Hamilton Anxiety Scale (HAM-A) score between 6 and 24, and blood pressure ≤ 129/80 mmHg. Exclusion criteria included: current use of psychoactive medications, including selective serotonin reuptake inhibitors (SSRIs), serotonin norepinephrine reuptake inhibitors (SNRIs), benzodiazepines, tricyclic antidepressants, pregabalin, gabapentin, quetiapine, or buspirone; use of weight-modifying medications or nutritional supplements; ongoing psychological therapy; diagnosed depressive disorder, bipolar disorder, eating disorders, renal disease, hepatic disease, cancer, thyroid disease, or olive allergy. Participants were eliminated from the study if adherence to supplementation was below 70%, pregnancy occurred during the intervention period, or voluntary withdrawal from the study occurred.

### 2.4. Sample Size Calculation

Sample size estimation was performed using OpenEpi (Open source epidemiologic statistics for public health (Version 3.01). https://www.openepi.com) based on IL-6 changes reported by [33] Javadi et al. (2019) following olive leaf polyphenol supplementation. Using mean differences reported between experimental and control groups, the estimated sample size was 29 participants per group. Due to limited participant availability and strict inclusion criteria, the planned sample size was not fully achieved. Therefore, this study should be considered exploratory and a pilot report.

### 2.5. Randomization, Allocation Concealment, and Blinding

Participants were randomly assigned to the OLE or Pb group using a permuted block randomization procedure generated with Random Allocation Software 2.0 (Random Allocation Software; Mahmood Saghaei, available from https://random-allocation-software.software.informer.com, accessed on 1 October 2024). Allocation concealment was maintained through coded supplement containers prepared by independent collaborators not involved in participant assessment, laboratory analyses, or statistical procedures. OLE and placebo capsules were packaged in identical bottles with similar appearance, labeling, and presentation. To prevent identification of treatment allocation, we used the same bottles for both treatments. Since OLE has a particular odor, it was stored in the bottles used for placebo for at least two weeks to ensure that the odor impregnated the bottle. After this period OLE was taken out and Pb was put in. Then, the bottle was considered ready to be handed to a participant. Capsules were the same size, but the color in OLE was transparent and Pb dark green (both colors used in commercial presentations). The number of capsules in Pb was 2 to 3 more than that of OLE, and new capsules with new bottles were given every two weeks. The study followed a double-blind design. Participants, clinical evaluators, laboratory personnel, and investigators responsible for outcome assessment and statistical analyses remained blinded to group allocation throughout the intervention and data analysis phases. Supplement coding, treatment assignment, and capsule distribution were performed by independent social service personnel and doctoral students affiliated with the research group who had no participation in psychological evaluations, biochemical analyses, or statistical interpretation. Treatment codes were revealed only after completion of all statistical analyses. To reduce detection and performance bias, anxiety assessments were performed using standardized administration procedures, and laboratory analyses were conducted according to manufacturer protocols without access to participant group information.

### 2.6. Intervention

Participants assigned to the intervention group received one daily capsule containing 750 mg of olive leaf extract (Nutricost^®^, Nutricost Manufacturing LLC, Vineyard, UT, USA) standardized to 20% oleuropein. Participants in the placebo group received one daily capsule containing 100 mg of food grade silica, which was selected because silica was also used as an excipient in the OLE formulation. Participants were instructed to consume one capsule daily after breakfast for 12 consecutive weeks. No dietary modifications or caloric restriction interventions were prescribed during the study period to minimize behavioral confounding effects.

### 2.7. Anxiety Symptomatology Assessment

Anxiety symptomatology was evaluated using the Hamilton Anxiety Scale (HAM-A), Beck Anxiety Inventory (BAI), and State-Trait Anxiety Inventory (STAI). To reduce familiarization effects and response bias, psychological scales were alternated throughout follow-up evaluations according to the experimental timeline. HAM-A assessments were additionally subdivided into psychic and somatic anxiety subscales for secondary analyses. All psychological evaluations were conducted by trained evaluators blinded to treatment allocation.

### 2.8. Anthropometric and Body Composition Assessment

Weight, lean mass percentage, and fat mass percentage were measured using a bioimpedance scale (Omron^®^ HBF-514C Healthcare Co., Ltd., Kyoto, Japan). Participants attended evaluations with a minimum fasting period of 4 h. Blood pressure was measured using an automated sphygmomanometer (Omron^®^ HEM-7120 Healthcare Co., Ltd., Kyoto, Japan) following standardized procedures.

### 2.9. Nutritional Assessment and Adherence Monitoring

A 24 h dietary recall was conducted twice monthly, including one weekday and one weekend day, to estimate caloric intake and macronutrient distribution. Nutritional analysis was performed using the Mexican System of Equivalent Foods (4th edition). Treatment adherence was monitored biweekly through capsule counting and participant self-report. Participants with adherence lower than 70% were excluded from final analysis.

### 2.10. Biochemical Analyses

Venous blood samples were collected at baseline and after the 12-week intervention period following overnight fasting conditions. Participants were instructed to not do exercise or consume caffeine for at least 24 h before sample collection. Alcohol consumption was instructed to be restricted for at least 72 h. Serum was separated by centrifugation at 3500 rpm for 10 min and stored at −25 °C until biochemical analysis. Circulating concentrations of interleukin 6 (IL 6), tumor necrosis factor alpha (TNF α), cortisol, leptin, and insulin were quantified using commercially available ELISA kits according to the manufacturers’ instructions, including the Legend Max™ Human IL 6 kit (Cat. 430507), Legend Max™ Human TNF α kit (Cat. 430207), Invitrogen™ Cortisol Competitive kit (EIAHCOR), Invitrogen™ Human Leptin kit (KAC2281), and Invitrogen™ Insulin ELISA kit (KAQ1251). Absorbance readings were obtained using an Agilent EPOCH2NS microplate reader. Total cholesterol, triglycerides, HDL cholesterol, and high-sensitivity C-reactive protein (hs CRP) concentrations were determined using spectrophotometric or turbidimetric methods with a Mindray BS 120 analyzer, whereas LDL cholesterol levels were estimated using the Friedewald equation. All sample processing and biochemical analyses were performed by laboratory personnel blinded to group allocation throughout the study.

### 2.11. Statistical Analysis

Descriptive data are presented as median and interquartile range (IQR). The Mann–Whitney Test was performed to evaluate differences between groups, and the Wilcoxon Test was used for within-group analysis. Spearman correlation analyses were performed to evaluate associations between psychological, inflammatory, metabolic, and anthropometric variables. Statistical significance was established at *p* < 0.05. All statistical analyses were performed using GraphPad Prism version 8.0 (GraphPad Software, San Diego, CA, USA).

## 3. Results

### 3.1. Sample Size and Demographics Analysis

**Figure 2:**
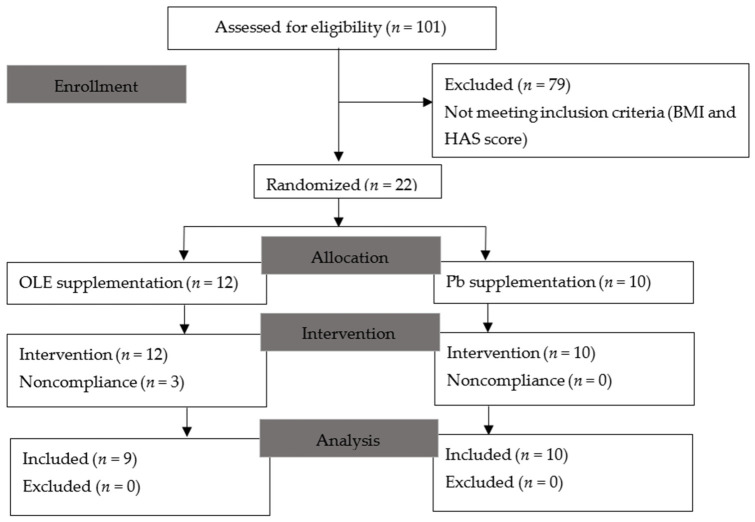
CONSORT flow diagram. Number of subjects assessed since the promotion of the study up to the conclusion, including excluded subjects. OLE: olive leaf extract; Pb: placebo; TNF-α: tumor necrosis factor alpha; hs-CRP: high-sensitivity C-reactive protein.

The target sample size was not achieved due to limited participant recruitment and availability. The study was promoted from October 2024 to November 2025. In total 101 subjects applied for entrance. Only 26 were able to pass the first filter and only 22 met all the inclusion criteria. In the end, 19 subjects completed the treatment period, and 3 ended up leaving because they were unable to stick to the supplementation due to their personal activities, usually forgetting to take the capsule; no participant reported adverse effects from taking any of the components (Figure 2). People not included with HAM-A scores higher than required were suggested to attend a free mental health service provided within CUCS.

Information on baseline subject demographics is shown in Table 1. Mann–Whitney analysis was made for each inclusion criteria variable with no statistically significant difference between groups (*p* < 0.05).

### 3.2. Anxious Symptomatology Analysis

Our first goal was to evaluate OLE effects in anxious symptomatology. For this, we used three different scales (HAM-A, BAI and STAI). The Hamilton Anxiety Scale (HAM-A) showed no significant differences between groups either at baseline (OLE 14 (12–18) vs. Pb 13 (8–16.75), *p* = 0.32, U = 32.5) or at the end (OLE 9 (3.5–14.5) vs. Pb 7 (5.75–8.5), *p* = 0.95, U = 44). Within groups a significant reduction in HAM-A score, OLE baseline vs. OLE end (*p* = 0.011, W = −41.00) and Pb baseline vs. Pb end (*p* = 0.011, W = −42.00) was observed. Later, we divided this score into two categories of the scale, psychic and somatic anxiety. Analysis between groups showed no significant difference at baseline nor at the end in psychic anxiety: OLE baseline 10 (8.5–12) vs. Pb baseline 7 (5.25–10.5), *p* = 0.10, U = 25; OLE end 5, IQR 7 (2.5–9.5) vs. Pb end 5 (3–6.75), *p* = 0.98, U = 44, respectively. Wilcoxon analysis showed significant diminished scores in both groups, OLE baseline vs. OLE end (*p* = 0.007, W = −36) and Pb baseline vs. Pb end (*p* = 0.04, W = −31). For somatic anxiety no statistically significant difference was seen in either measure: OLE baseline 5 (2.5–7.5) vs. Pb baseline 5 (2.75–7.25), *p* = 0.89, U = 43 and OLE end 3 (0–5) vs. Pb end 2 (1–3.25), *p* = 0.95, U = 44. Within groups somatic anxiety was reduced in Pb (*p* = 0.02, W = −38).

**Figure 3:**
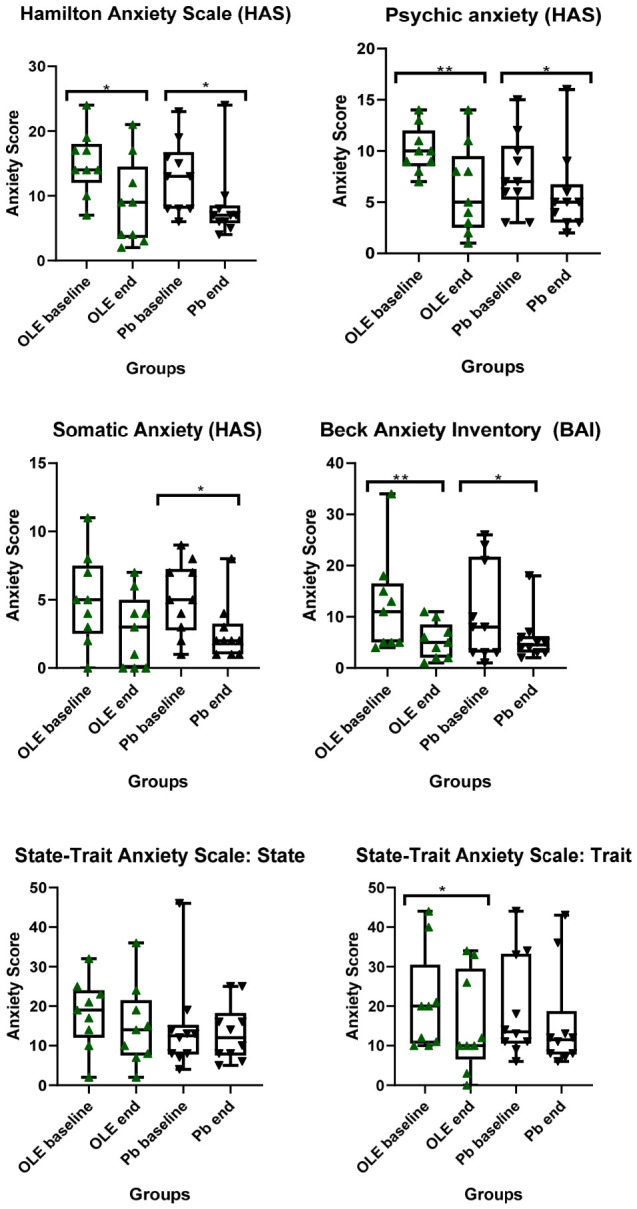
Anxiety score for different scales. BAI: Beck Anxiety Inventory; OLE: olive leaf extract; Pb: placebo. Values are presented in median with min. to max. (* *p* < 0.05; ** *p* < 0.01).

For the Beck Anxiety Inventory (BAI) no statistical difference was observed between groups (OLE baseline 11 (5–16.5) vs. Pb baseline 8 (3–21.75), *p* = 0.48, U = 36; OLE end 5 (2–8.5) vs. 4 (3–6.25), *p* = 0.9, U = 45). Within both groups, statistically significant differences were seen: OLE baseline vs. OLE end (*p* = 0.0078, W = −36) Pb baseline vs. Pb end (*p* = 0.03, W = −36). For State-Trait Anxiety, in the State and Trait anxiety category, no statistically significant differences were observed between groups: STAI-State, OLE baseline 19 (12–24) vs. Pb baseline 12.5 (7.75–15.25), *p* = 0.12, U = 26; OLE end 14 (7.5–21.5) vs. Pb end 12 (7.5–18.25), *p* = 0.88, U = 43, STAI-Trait, OLE baseline 20 (10.5–30.5) vs. Pb baseline 13.5 (10.5–33.25), *p* = 0.67, U = 39.5; OLE end 10 (6.5–29.5) vs. Pb end 11.5 (7.75–18.75), *p* = 0.82, U = 42. Within-group analysis showed a reduction in STAI-Trait score only in the OLE group (*p* = 0.02, W = −37) (Figure 3).

### 3.3. Inflammatory and Metabolic Parameter Analysis

**Figure 4:**
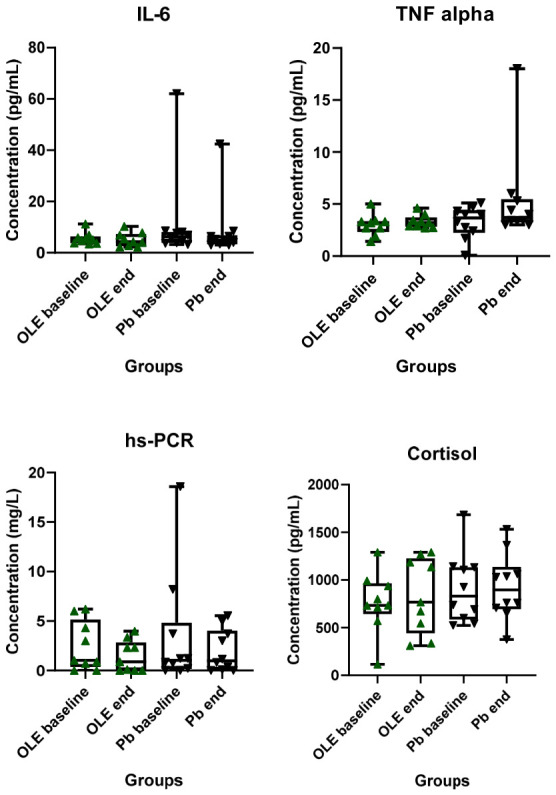
Inflammatory parameters. IL-6: Interleukin 6; hs-CRP: high-sensitivity C-reactive protein; OLE: olive leaf extract; Pb: placebo. Values are presented in median and IQR.

Because of their association with anxiety, we also evaluated some inflammatory parameters (IL-6, TNF-α, hs-CRP and cortisol). These were taken at baseline and after the three-month supplementation. No statistically significant difference was observed between or within groups in either of them: IL-6, OLE baseline 5.1 (3.75–6.4) vs. Pb baseline 6.3 (3.8–8.15), *p* = 0.48, U = 36; OLE end 4.3 (2.55–7.35) vs. Pb end 4.95 (3.32–6.9), *p* = 0.64, U = 39. TNF-α, OLE baseline 3.2 (2.3–3.3) vs. Pb baseline 3.6 (2.22–4.4), *p* = 0.48, U = 36; OLE end 3.2 (2.8–3.7) vs. Pb 3.7 (3.24–5.47), *p* = 0.06, U = 22; within groups Pb baseline vs. Pb end, *p* = 0.071, W = 31; OLE baseline vs. OLE end, *p* = 0.44, W = 14. Hs-CRP, OLE baseline 1 (0.3–5) vs. Pb baseline 1 (0.15–4.8), *p* = 0.95, U = 44; OLE end 0.87 (0–2.8) vs. Pb end 0.97 (0.05–4.01), *p* = 0.59, U = 38; within groups Pb baseline vs. Pb end, *p* = 0.57, W = −11; OLE baseline vs. OLE end, *p* = 0.21, W = −13. Cortisol, OLE baseline 734 (642–965) vs. Pb baseline 831 (586–1135), *p* = 0.71, U = 40; OLE end 767 (440–1228) vs. Pb end 896 (694–1138), *p* = 0.70, U = 40 (Figure 4).

**Figure 5:**
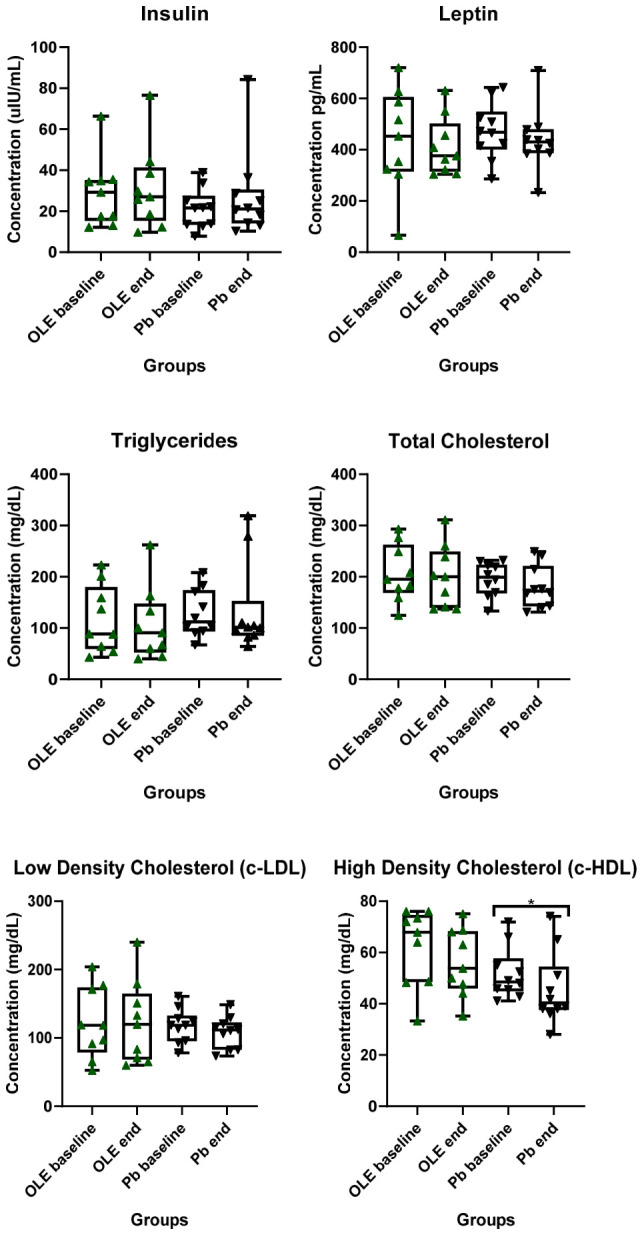
Metabolic parameters. OLE: olive leaf extract; Pb: placebo. Values are presented in median with min. and max. (* *p* < 0.05).

For exploratory outcomes, we also analyzed some metabolic parameters linked to anxiety like insulin, leptin and lipid profile (TGs, TC, c-HDL and c-LDL). No statistically significant differences were found between nor within groups, except in c-HDL where a significant reduction was observed in the Pb group from baseline to end: Insulin, OLE baseline 29.3 (15.28–35.20) vs. Pb baseline 21.60 (13.4–27.5), *p* = 0.40, U = 34; OLE end 27 (15.4–41.37) vs. Pb end 21.10 (14–30.63), *p* = 0.49, U = 36. Leptin, OLE baseline 453 (314–607) vs. Pb baseline 468 (400–549), *p* = 0.78, U = 41; OLE end 376 (314–503) vs. Pb end 430.5 (386–480), *p* = 0.40, U = 34. Triglycerides, OLE baseline 88.4 (59–180) vs. Pb baseline 112 (93.25–174), *p* = 0.40, U = 34; OLE end 90.9 (52–148) vs. Pb end 101 (85–153), *p* = 0.35, U = 33. Total Cholesterol, OLE baseline 734 (642–965) vs. Pb baseline 831 (586–1135), *p* = 0.72, U = 40; OLE end 767 (440–1228) vs. Pb end 895.5 (694–896), *p* = 0.70, U = 40. c-HDL, OLE baseline 68 (48–75) vs. Pb baseline 49 (45–58), *p* = 0.07, U = 23; OLE end 54 (46–68) vs. Pb end 40 (38–55), *p* = 0.1128, U = 25; Pb baseline vs. Pb end (*p* = 0.02, W = −44). C-LDL, OLE baseline 119 (79–174) vs. Pb baseline 119 (95–133), *p* = 0.95, U = 44; OLE end 120 (68–165) vs. Pb end 112 (83–123), *p* = 0.78, U = 41 (Figure 5).

### 3.4. Weight, Fat and Lean Mass Percentage Analysis

**Figure 6:**
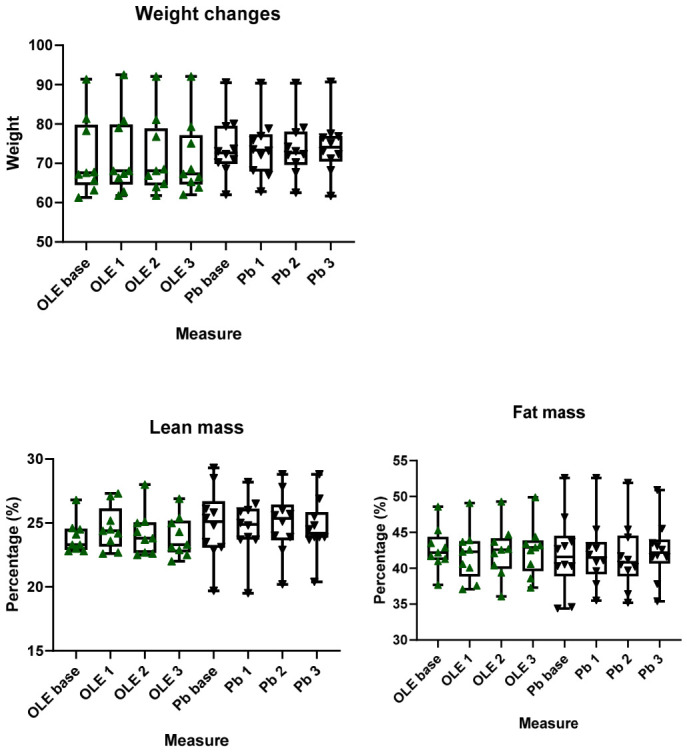
Weight, Lean and Fat mass. No statistically significant difference was shown. OLE base: olive leaf extract baseline; Pb base: Placebo baseline. Values are presented in median with min. and max.

These parameters were evaluated to assess possible changes that may be associated with the inflammatory state in excess weight. No significant differences were found between or within groups from baseline until the end. We present the comparison between the baseline and end: Weight, OLE baseline 67.6 (64.45–79.85) vs. Pb baseline 72.65 (69.80–79.55), *p* = 0.27, U = 31; OLE end 67.30 (64.60–77.20) vs. Pb end 74.10 (70.45–76.98), *p* = 0.28, U = 31.50. Lean mass, OLE baseline 23.3 (22.9–24.5) vs. Pb baseline 25.1 (23–26.7), *p* = 0.23, U = 30; OLE end 24.4 (23.15–26.15) vs. Pb end 24.9 (23.7–26.13), *p* = 0.64, U = 39. Fat mass, OLE baseline 42.2 (41.15–44.4) vs. Pb baseline 41.6 (38.8–44.5), *p* = 0.58, U = 38 (Figure 6).

### 3.5. Twenty Four-Hour Nutritional Recall Analysis

**Figure 7:**
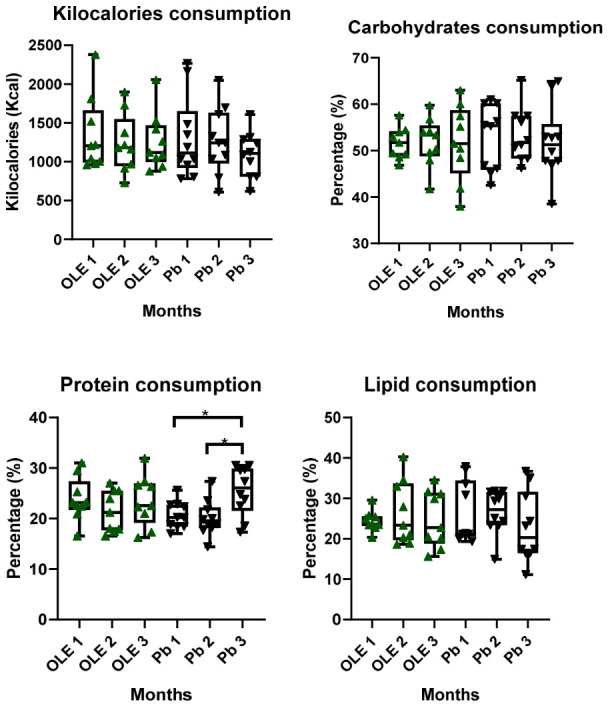
Twenty-four-hour nutritional recall. Kilocalories, carbohydrates, protein and fat consumption monthly. OLE: olive leaf extract; Pb: Placebo. Values are presented in median with min. and max. (* *p* < 0.05).

In case we found changes in anthropometric measures, we conducted a 24 h nutritional recall twice a month (one time referring to a midweek and the other time a weekend day) to assess a possible nutritional cause. From these nutritional recalls, kilocalories and macronutrient (carbohydrates, proteins and fat) percentage ingestion were calculated and synthetized as monthly consumption. No differences between groups were found. Within groups there was a significant elevation of protein consumption in Pb between the baseline and month 2 with the end measure: Kilocalories, OLE baseline 1206 (988–1665) vs. Pb baseline 1113 (919.9–1654), *p* = 0.58, U = 38; OLE end 1120 (994.5–1470) vs. Pb end 1108 (807–1294), *p* = 0.48, U = 36. Carbohydrates, OLE baseline 51.7 (48.8–54.18) vs. Pb baseline 55.35 (45.85–60.19), *p* = 0.58, U = 38; OLE end 51.5 (45.13–58.7) vs. Pb end 51.28 (47.52–55.7), *p* = 0.64, U = 39. Protein, OLE baseline 23.15 (21.6–27.38) vs. Pb baseline 20.23 (18.4–22.74), *p* = 0.07, U = 23; OLE end 22.5 (19.15–26.97) vs. Pb end 26.05 (21.51–29.92), *p* = 0.32, U = 32.5. Wilcoxon analysis within Pb baseline vs. Pb end, *p* = 0.048, W = 39; Pb month 2 (19.38 (18.10–22.20) vs. Pb end (*p* = 0.01, U = 39). Fat, OLE baseline 23.6 (23.13–25.6) vs. Pb baseline 21.23 (20.49–34.45), *p* = 0.70, U = 40; OLE end 22.75 (18.75–31.38) vs. Pb end 20.33 (16.45–31.64), *p* = 0.70, U = 40 (Figure 7).

Finally, Spearman correlation was made within groups. For the Pb group, end HAM-A score correlated positively with weight at baseline (*p* = 0.018, r = 0.739), month 1 (*p* = 0.034, r = 0.638), month 2 (*p* = 0.029, r = 0.695) and end (*p* = 0.043, r = 0.659); with end STAI-state (*p* = 0.035, r = 0.677) and end STAI-Trait (*p* = 0.004, r = 0.830). End psychic anxiety (HAM-A) was related to weight at baseline (*p* = 0.005, r = 0.819), month 1 (*p* = 0.003, r = 0.843), month 2 (*p* = 0.001, r = 0.905) and end (*p* = 0.006, r = 0.812); end STAI-Trait (*p* = 0.042, r = 0.659) and baseline BAI (*p* = 0.005, r = 0.822). End somatic HAM-A was associated with end cortisol (*p* = 0.027, r = 0.702). End BAI correlated to weight at baseline (*p* = 0.003, r = 0.856), month 1 (*p* = 0.004, r = 0.832), month 2 (*p* = 0.004, r = 0.832) and end (*p* = 0.006, r = 0.813); to end STAI-Trait (*p* = 0.012, r = 0.766). Baseline STAI-Trait was associated with baseline BMI (*p* = 0.011, r = 0.778), baseline BAI (*p* = 0.011, r = 0.775) and somatic HAM-A (*p* = 0.047, r = 0.648. End STAI-Trait was related to end HAM-A (*p* = 0.004, r = 0.830), end psychic anxiety (*p* = 0.042, r = 0.659) and end BAI (*p* = 0.012, r = 0.766). hs-CRP correlated moderately with baseline STAI-Trait (*p* = 0.077, r = 0.591).

**Figure 8:**
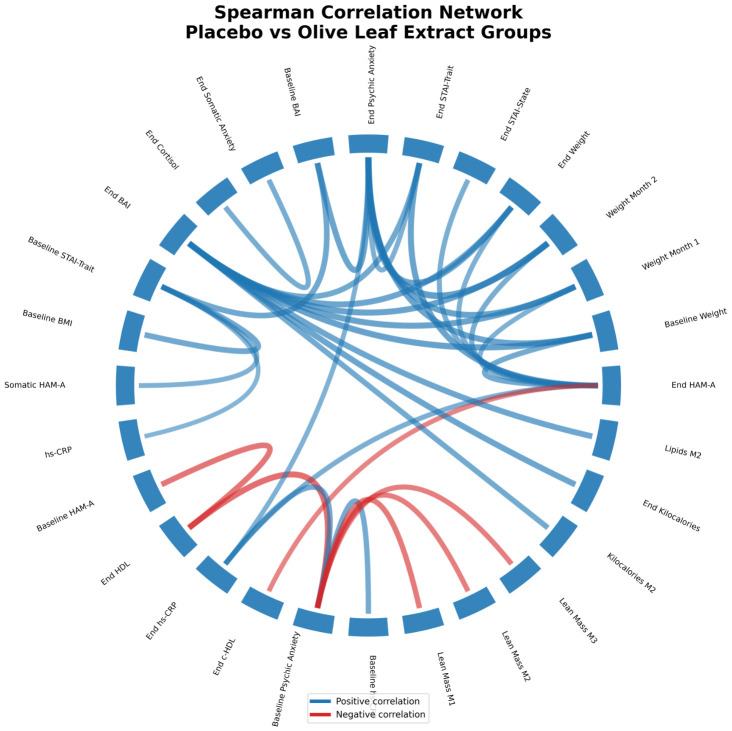
Correlation network diagram illustrating significant Spearman correlations among anxiety symptomatology, inflammatory biomarkers, lipid profile parameters, and body composition variables in the olive leaf extract and placebo groups. Blue connections indicate positive correlations, whereas red connections indicate negative correlations. Significant associations were observed between HAM-A anxiety scores, hs-CRP, IL-6, triglycerides, fat mass, and lean mass parameters. Only statistically significant correlations are displayed (*p* < 0.05).

For the OLE group, baseline HAM-A correlated to end HDL (*p* = 0.020, r = −0.766). End HAM-A had a moderated correlation to end hs-CRP (*p* = 0.053, r = 0.671) and negatively to end c-HDL (*p* = 0.071, r = −0.639). Baseline psychic anxiety was associated with end HDL (*p* = 0.008, r = −0.832), baseline hs-CRP (*p* = 0.031, r = 0.726), end hs-CRP (*p* = 0.026, r = 0.743) and lean mass percentage at month 1 (*p* = 0.030, r = −0.731), month 2 (*p* = 0.046, r = −0.689), and month 3 (*p* = 0.046, r = −0.689). End psychic anxiety correlated moderately with end hs-CRP (*p* = 0.070, r = 0.639). End BAI was associated with kilocalories consumption at month 2 (*p* = 0.027, r = 0.745) and end (*p* = 0.011, r = 0.812); to lipid consumption at month 2 (*p* = 0.038, r = 0.803) (Figure 8).

## 4. Discussion

The present randomized double-blind placebo-controlled pilot trial evaluated the effects of OLE supplementation on anxiety symptomatology, inflammatory markers, and metabolic parameters in women with excess weight. Because the planned sample size was not achieved, the present findings should be interpreted as preliminary. Overall, no significant effects of OLE supplementation compared with placebo were observed. Therefore, our primary hypothesis was not supported.

Although no significant effects of OLE supplementation compared with placebo were observed, the within-group changes identified in this pilot study may provide useful information for future investigations evaluating the potential effects of OLE on anxiety symptomatology.

In the placebo group, significant reductions were observed between the baseline and the end of the intervention in HAM-A total score, psychic anxiety, somatic anxiety, BAI score, c-HDL levels, and protein consumption. Spearman correlation analysis revealed that end HAM-A, psychic anxiety, and BAI scores were positively associated with body weight and end STAI-Trait scores. In addition, somatic anxiety was positively correlated with end cortisol levels and a tendency toward increased TNF-α was shown.

In the OLE group, significant reductions were observed in HAM-A total score, STAI-Trait score, psychic anxiety, and BAI score, with greater reductions in psychic anxiety and BAI scores compared with those observed in the placebo group. Correlation analyses showed that baseline psychic anxiety was positively associated with baseline and end hs-CRP concentrations and negatively associated with end c-HDL levels. Furthermore, end HAM-A scores were moderately positively correlated with hs-CRP and negatively correlated with c-HDL levels, whereas psychic anxiety showed a moderate positive association with hs-CRP. These findings suggest that anxiety symptomatology, inflammatory status, and lipid metabolism may be interrelated in women with excess weight, although the cross-sectional nature of these correlations precludes causal inference.

An additional observation was that reductions within the OLE group appeared more pronounced for psychic anxiety than for somatic anxiety. Psychic anxiety primarily reflects cognitive and emotional symptoms, including excessive worry, apprehension, and mental tension, whereas somatic anxiety and BAI scores predominantly capture autonomic and physical manifestations of anxiety [39]. Although no statistically significant changes were detected in inflammatory biomarkers, participants receiving OLE showed moderate associations between anxiety scores and hs-CRP, together with the absence of the tendency toward increased TNF-α observed in the placebo group. Previous studies have shown that pro-inflammatory cytokines, particularly TNF-α, may increase blood–brain barrier permeability and promote neuroimmune signaling within limbic structures such as the amygdala and hippocampus [40,41], regions critically involved in emotional processing and anxiety regulation. Considering the anti-inflammatory properties of olive polyphenols, it is plausible that OLE supplementation may preferentially influence cognitive-affective dimensions of anxiety through modulation of neuroinflammatory pathways. However, given the exploratory nature and limited statistical power of this pilot trial, these mechanistic interpretations should be considered hypothesis-generating rather than causal.

Based on the correlations and guided by the different changes observed within each group, it is possible that the reduction in anxious symptomatology in Pb may be due to a placebo effect. First, the graphical patterns in Pb are more irregular than in OLE. Secondly, BAI refers mostly to neurophysiological (numbness, dizziness, wobbliness, trembling, and unsteadiness), autonomic (feeling hot, indigestion, and sweatiness) and panic (heart pounding, feeling of choking, breathing difficulty, and fear of dying) aspects of anxiety. These processes are related to the somatic measures in HAM-A (somatic-muscular, respiratory, cardiovascular, gastrointestinal, genitourinary and autonomic items) [39]. It has been observed that when certain suggestions of a specific treatment match the personal experience of an individual, a placebo effect may be enhanced and influence cardiovascular, gastrointestinal and pulmonary functions [42]. Thus, the expectations of the participants in reducing anxious symptomatology and their previous experiences of that may have contributed to the reduction observed within the Pb group. Also, open-labeled placebos have shown the capacity to reduce cortisol levels [43]. In line with this, end cortisol levels correlated with end somatic anxiety, for which statistically significant reduction was only shown in the Pb group. Also, there was an increase in protein consumption and a tendency toward reduced lipid consumption in Pb. Since these patterns are associated with improved lipid profile, the reduction in c-HDL in Pb opens the possibility of being due to the pro-inflammatory state; however, other variables that were not assessed, like protein and lipid quality consumption, can also be associated with this phenomenon.

In contrast, OLE showed a greater reduction in end psychic anxiety and BAI; also, a significant reduction in STAI-Trait that was not observed in Pb. HAM-A scale is associated with GAD. Trait anxiety also correlates to GAD and can reflect current anxious symptomatology [44]. Compared to Pb the greater reduction in these parameters may be due to the association of the psychic aspects in each of these scales, BAI (unable to relax, fearful of the worst, terrified, nervous, and scared) and HAM-A (anxious mood, tension, fearfulness, cognitive function, and depressed mood). In concordance with this is the correlation in Pb between end BAI and end psychic anxiety with end STAI-Trait. In these three parameters the significance was different within OLE and Pb.

For the inflammatory and metabolic parameters, Pb presents a tendency of increased TNF-α and significant reduction in c-HDL levels. This lipoprotein has antioxidant and anti-inflammatory functions [45]. The cholesterol efflux ability in c-HDL inhibits M1 polarization in macrophages [46]. Inflammation can reduce c-HDL serum levels and alter its protective capacity [47]. In contrast, OLE showed a tendency of reduced hs-CRP. The main polyphenol in olive leaf, oleuropein, is the main responsible of the anti-inflammatory effect found in the extract. It can be hydrolyzed into hydroxytyrosol and oleuropein aglycone, among others [48]. Hs-CRP can be enhanced by reactive oxygen species [49]. The antioxidant effect from the extract can influence its synthesis; however, this effect is not consolidated since studies that measured the effect of OLE in CRP are heterogeneous [50,51]. Another correlation with anxiety found in OLE was c-HDL. It has been observed that polyphenols from olive oil (like hydroxytyrosol and oleuropein) can improve c-HDL cholesterol efflux and its anti-inflammatory effect [52]. Also, in OLE the elevated tendency toward end TNF-α levels is not observed. The extract is also able to inhibit its synthesis by altering the NF-κβ pathway [53,54,55]. The behavior of these parameters in the OLE group may in part be explanatory of the anxious symptomatology phenotype changes seen in this group.

In Pb a correlation was observed between weight and anxious symptomatology signaling, in part, the association between metainflammation and anxiety. Also, end HAM-A, end psychic anxiety and end BAI were correlated with end STAI-Trait; however, only the first two scales had a significant difference between the baseline and end. In addition, Pb showed a reduction in somatic anxiety that was not found in OLE and a correlation with end cortisol. This phenotype of anxious symptoms can be more easily associated with a possible placebo effect involving the autonomic nervous system that has been observed in multiple studies.

Placebo responses are particularly robust in anxiety and other psychiatric clinical trials, where expectancy, therapeutic interaction, and repeated psychological assessments frequently produce clinically meaningful symptom improvements. Consistent with this phenomenon, both groups in the present study exhibited reductions in several anxiety measures. Therefore, part of the observed improvement may reflect non-specific placebo-related effects. Nevertheless, the OLE group showed a distinct pattern characterized by greater reductions in psychic anxiety and STAI-Trait scores, together with the absence of the tendency toward increased TNF-α observed in the placebo group. Although preliminary, these differential findings suggest that OLE supplementation may exert effects beyond expectancy-related responses and warrant confirmation in larger randomized controlled trials.

Based on the correlations observed in the Pb group between these three scales and the different results compared with OLE group we believe that a possible effect from the extract in these parameters is plausible. In line with this possibility are the tendencies observed in hs-CRP and TNF-α and the changes in end c-HDL. The Pb group showed a tendency toward increased TNF-α and less c-HDL levels, conditions that can reflect greater inflammation. In contrast, OLE showed a tendency toward reduced hs-CRP, and the small increase in TNF-α was not observed. The correlations between HAM-A and psychic anxiety with hs-CRP and c-HDL in the OLE group, and the possibility of olive leaf extract polyphenols improving these parameters, reflecting diminished inflammation, suggests a possible link between the administration of OLE and the reduction in anxious symptomatology through pathways associated with inflammation.

Future randomized controlled trials should be adequately powered to detect clinically meaningful changes in anxiety symptomatology and inflammatory biomarkers following OLE supplementation. In addition to increasing sample size, future studies should consider extending the intervention period and incorporating multiple follow-up assessments to better characterize the temporal dynamics of psychological and biological responses.

Given the exploratory associations observed between anxiety measures, inflammatory markers, and c-HDL levels, future investigations should adopt a more comprehensive phenotyping approach. This could include the assessment of additional biomarkers involved in neuroimmune and metabolic regulation, such as IL-1β, oxidative stress markers, adipokines, and measures of HDL functionality rather than solely HDL concentration. Simultaneous evaluation of HPA axis activity through repeated cortisol measurements may also provide mechanistic insight into the relationship between inflammation, stress, and anxiety. Additionally, strategies should be incorporated to characterize and control expectancy effects, including the assessment of treatment expectations and participant beliefs regarding supplementation. Moreover, stratification according to baseline anxiety severity, inflammatory status, or metabolic profile may help identify subpopulations that could derive greater benefit from OLE supplementation.

Finally, integrating multi-domain approaches combining psychological scales, dietary assessments, inflammatory biomarkers, and metabolomic or microbiome analyses may contribute to elucidating the biological pathways through which olive polyphenols influence mental health outcomes. Such approaches could facilitate the identification of predictive biomarkers and support the development of personalized nutritional interventions targeting anxiety symptoms in individuals with excess weight.

## 5. Conclusions

Due to the limited sample size and the pilot nature of this study, our findings should be considered preliminary. OLE supplementation did not produce significant effects compared with placebo on anxiety symptomatology, inflammatory markers, or metabolic parameters. Nevertheless, distinct patterns of change were observed within each group, suggesting that specific psychological, inflammatory, and metabolic variables may be relevant for future investigations. Although these findings do not allow conclusions regarding the efficacy of OLE, they provide valuable information for the design of adequately powered clinical trials aimed at exploring the potential relationship between olive leaf polyphenols, inflammation, and anxiety-related outcomes.

## Figures and Tables

**Table 1:** Mann–Whitney test of entry data. Pb: placebo; OLE: olive leaf extract; BMI: Body Mass Index; HAM-A: Hamilton Anxiety Scale. Values are presented in median with IQR (*p* < 0.05).

Variables	Pb (*n* = 10)	OLE (*n* = 9)	*p* Value	U Value
Age (years)	26.30 (19.75–32)	25.67 (20–31.5)	0.9	45
Height (meters)	1.63 (1.58–1.68)	1.62 (1.58–1.64)	0.34	33
Weight (kg)	72.65 (69.8–79.55)	67.60 (64.45–79.85)	0.28	31
BMI (kg/m^2^)	26.95 (26.5–28.33)	26 (25.35–28.95)	0.33	32.5
Systolic pressure (mmHg)	106 (102–115)	112 (106–117)	0.34	33
Diastolic pressure (mmHg)	69.5 (67–78)	76 (68–79)	0.48	36
HAM-A	13 (8–16.75)	14 (12–18)	0.32	32.5

## Data Availability

The original contributions presented in this study are included in the article. Further inquiries can be directed to the corresponding authors.

## Abbreviations

BBB	Blood–brain barrier
GAD	General anxiety disorder
CRH	Corticotropin-releasing hormone
HPA	Hypothalamic–Pituitary–Adrenal axis
NMDA	N-methyl-D-aspartate
BDNF	Brain-derived neurotrophic factor
PVN	Paraventricular Nucleus
VAT	Visceral adipose tissue
TLR	Toll-like receptor
Hs-CRP	High-sensitivity C-reactive protein
IR	Insulin receptor
KO	Knockout
VTA	Ventral tegmental area
OLE	Olive leaves extract
GMA	Guadalajara Metropolitan Area
UdeG	Universidad de Guadalajara
CUCS	Centro Universitario de Ciencias de la Salud
HAM-A	Hamilton Anxiety Scale
BAI	Beck Anxiety Inventory
STAI	State-Trait Anxiety Inventory
BMI	Body Mass Index
SSRI	Selective serotonin reuptake inhibitor
SNRI	Serotonin and norepinephrine reuptake inhibitor
S.E.M	Standard Error of the Mean
TG	Triglyceride
FFA	Free fatty acid
PBMC	Peripheral Blood Mononuclear Cell
AMPAR	Alpha-amino-3-hydroxy-5-methyl-4-isoxazolepropionic acid receptor
PTSD	Post-traumatic stress disorder
